# UAV-based multitier feature selection improves nitrogen content estimation in arid-region cotton

**DOI:** 10.3389/fpls.2025.1639101

**Published:** 2025-08-12

**Authors:** Fengxiu Li, Chongqi Zhao, Yingjie Ma, Ning Lv, Yanzhao Guo

**Affiliations:** ^1^ College of Hydraulic and Civil Engineering, Xinjiang Agricultural University, Urumqi, China; ^2^ Xinjiang Key Laboratory of Hydraulic Engineering Safety and Water Disaster Prevention, Urumqi, China; ^3^ Key Laboratory of North-west Oasis Water-Saving Agriculture, Ministry of Agriculture and Rural Affairs, Xinjiang Academy of Agricultural and Reclamation Sciences, Shihezi, Xinjiang, China

**Keywords:** cotton, nitrogen, multispectral imagery, Elastic Net, Boruta-SHAP, machine learning

## Abstract

**Introduction:**

Nitrogen plays a pivotal role in determining cotton yield and fiber quality. Nevertheless, because high-dimensional remote-sensing data are inherently complex and redundant, accurately estimating cotton plant nitrogen concentration (PNC) from unmanned aerial vehicle (UAV) imagery remains problematic, which in turn constrains both model precision and transferability.

**Methods:**

Accordingly, this study introduces a hierarchical feature-selection scheme combining Elastic Net and Boruta–SHAP to eliminate redundant remote-sensing variables and evaluates six machine-learning algorithms to pinpoint the optimal method for estimating cotton nitrogen status.

**Results:**

Our findings reveal that five critical features (Mean_B, Mean_R, NDRE_GOSAVI, NDVI, GRVI) markedly enhanced model performance. Among the tested algorithms, random forest achieved superior performance (R² = 0.97–0.98; RMSE = 0.05–0.08), exceeding all alternatives. Both in-field observations and model outputs demonstrate that cotton PNC consistently decreases throughout development, but optimal conditions of 450 mm irrigation and 300 kg N ha⁻¹ sustain relatively elevated nitrogen levels.

**Discussion:**

Collectively, the study provides robust guidance for precision nitrogen management in cotton production within arid regions.

## 1 Introduction

Precision agricultural management is increasingly supported by the rapid advancement of information technology. The livelihoods of hundreds of millions of farmers worldwide are sustained by cotton, one of the world’s most important natural fiber crops (Hou et al., 2024). Nitrogen is regarded as a critical nutrient that determines cotton yield, fiber quality, and plant health. Consequently, accurate, real-time monitoring of the crop’s nitrogen status is essential for optimizing fertilization strategies, improving resource-use efficiency, and advancing sustainable agriculture (Li D. et al., 2024).

Accountingfor over 85 percent of China’s cotton output, Xinjiang stands as the nation’s premier cotton production base and plays a critical role in the global supply chain. Although the area benefits from plentiful light and heat, its extreme hydrothermal regime, characterized by just 150 to 200 millimeters of yearly rainfall and evaporation rates soaring to 2000 to 3000 millimeters, alters nitrogen transformation and loss relative to other production zones, thereby complicating precise nitrogen management (Zhou et al., 2023). Plastic film mulched drip irrigation can enhance water and fertilizer efficiencies, yet the intricate nitrogen migration and transformation dynamics within the beneath-film microenvironment mean that empirical fertilization practices are no longer sufficient for highly efficient large scale cultivation. At the same time, destructive sampling coupled with labor intensive laboratory analysis cannot deliver continuous real time data across space and time for field production. In recent years, unmanned aerial vehicle (UAV) remote sensing has been adopted as a powerful tool for crop nitrogen monitoring because of its efficiency, convenience, and non-destructive nature (Jia et al., 2025; Pei et al., 2023). Unmanned aerial vehicles equipped with multispectral cameras can capture abundant spectral and textural information. Previous studies have demonstrated that integrating spectral vegetation indices and texture features into machine-learning models enables accurate estimation of cotton plant nitrogen concentration (PNC) (Zhuang et al., 2024), Moreover, nitrogen-diagnosis accuracy at all potato growth stages has been significantly improved by an optimized texture index derived from UAV hyperspectral imagery (Fan et al., 2023). The considerable potential of integrating multi-source remote-sensing data with machine-learning algorithms for crop nitrogen estimation has been demonstrated by these studies.

However, the effectiveness and accuracy of current UAV-based approaches for cotton nitrogen assessment are still limited by several challenges. First, continuous nitrogen tracking is hindered by the tendency of vegetation indices to saturate during late growth stages. Second, although texture features have been introduced to complement spectral information, their potential has not yet been fully exploited (Wang et al., 2025). In addition, high collinearity and redundancy among multidimensional remote-sensing variables substantially reduce the predictive accuracy and generalizability of nitrogen-estimation models. Therefore, efficiently identifying key variables to build stable, high-performance models remains an urgent research priority.

In recent years, Elastic Net and Boruta–SHAP have gained widespread attention in the field of agricultural remote sensing due to their ability to effectively handle high-dimensional, collinear, and non-linear data. Elastic Net combines the advantages of L1 and L2 regularization, reducing redundancy among correlated variables while maintaining model interpretability. Previous studies have shown that using Elastic Net to predict crop nitrogen status and soil moisture content outperforms traditional methods in terms of stability and prediction accuracy (Cao et al., 2021; Yang et al., 2025). Boruta–SHAP combines importance measures with Shapley additive explanations, effectively capturing non-linear variable interactions. Recent agricultural applications have demonstrated that Boruta–SHAP excels in identifying key variables for maize nitrogen estimation and precision agriculture classification tasks, offering both high predictive performance and interpretability (Lu et al., 2025). Given that multi-level feature selection strategies balance statistical sparsity with model interpretability, they are particularly crucial for accurately estimating cotton nitrogen content. This study employs Elastic Net and Boruta–SHAP for feature selection. The integrated approach removes redundant features while retaining key variables involved in non-linear interactions, thus improving the robustness and interpretability of cotton nitrogen-estimation models.

This study aims to systematically compare the performance of six machine-learning algorithms—Bayesian-optimized Random Forest (RF), Gradient Boosting Decision Trees (GBDT), Extreme Gradient Boosting (XGB), Support Vector Regression (SVR), Kernel Ridge Regression (KRR), and Gaussian Process Regression (GPR)—in cotton nitrogen estimation, based on the optimized feature space obtained through Elastic Net and Boruta–SHAP feature selection, and to select the model with both the highest accuracy and the best generalizability. Ground-observed data will be combined to analyze the spatiotemporal distribution of cotton plant nitrogen and validate the model’s reliability, with the goal of providing efficient and interpretable technical support for precision fertilization decisions in cotton cultivation.

## 2 Materials and methods

### 2.1 Overview of the study area

**Figure 1:**
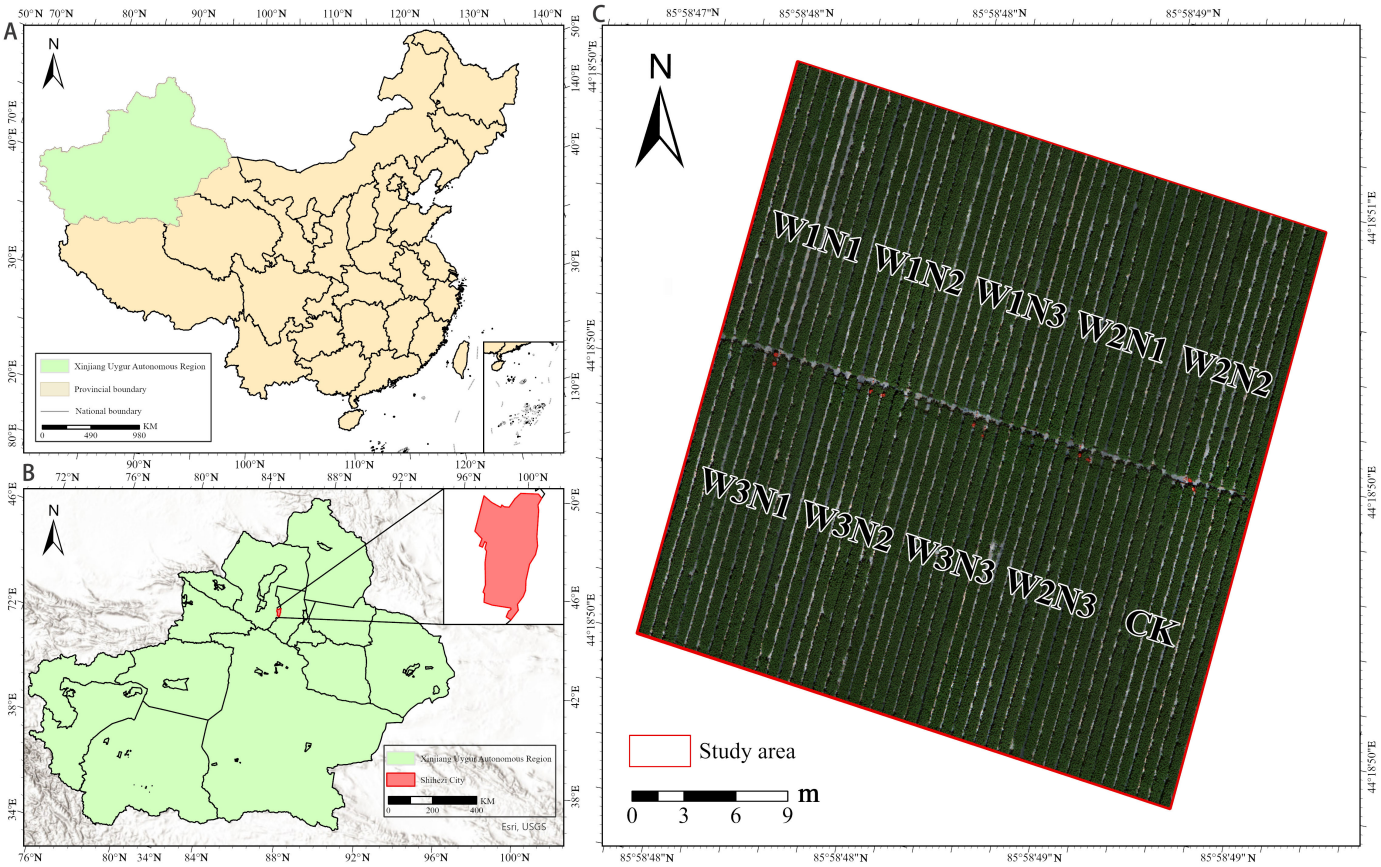
Study area and plot treatments. **(A)** Location of the Xinjiang Uygur Autonomous Region in China, **(B)** Location of Shihezi City within Xinjiang. **(C)** Experimental field layout showing water and nitrogen treatment plots (W1N1–W3N3) and the control (CK). The red border indicates the study area.

The study site lies in the Shihezi Reclamation Area of the Eighth Division, Xinjiang Production and Construction Corps (44°18′ N, 86° 03′ E; **Figure 1**). It experiences a typical arid to semi-arid continental climate, is topographically flat with a gentle south-east–to-north-west gradient, and sits at a mean elevation of 450 m. Annual sunshine duration reaches 2526–2874 h, whereas mean annual air temperature ranges between 6.5°C and 8.2°C. Soil at the site contains 11.9 g kg^-^¹ organic matter, 0.69 g kg^-^¹ total N, 37 mg kg^-^¹ available P, and 224 mg kg^-^¹ available K.

### 2.2 Experimental design

Based on a production survey of several farmer-managed cotton fields in the study area, on-farm irrigation volumes range from 350 to 550 mm and nitrogen inputs from 220 to 450 kg ha^-^¹ (Luo et al., 2024; Wan et al., 2024). These treatment levels were selected to provide adequate experimental contrast across the full range of practical farming conditions in the region. Accordingly, we implemented a two-factor water–nitrogen factorial experiment with three nitrogen rates—N1 (225 kg ha^-^¹), N2 (300 kg ha^-^¹) and N3 (375 kg ha^-^¹)—and three irrigation quotas—W1 (375 mm), W2 (450 mm) and W3 (525 mm)—plus a local-practice control (CK), yielding ten treatments in total, each replicated three times. Fertigation was applied nine times throughout the growing season; the detailed schedules are presented in **Table 1**, and irrigation-to-fertilizer ratios in **Table 2**. Uniform basal doses of phosphorus (540 kg ha^-^¹) and potassium (450 kg ha^-^¹) were supplied across all treatments. The field trial commenced with sowing on 21 April 2024 and concluded with harvest and yield assessment on 3 October 2024, thus covering the entire local cotton growing season. Each plot followed a “one mulch, three drip lines, six rows” configuration with a mulch width of 2.05 m, an intra-row spacing of 10 cm, alternating row spacings of 10 cm + 66 cm, and a planting density of 2641–000 plants ha^-^¹; border rows were established around the experiment. This design aligns with local water-fertilizer management practices, establishes clear contrasts in crop vigor among treatments, and provides a diverse, robust phenotypic dataset for developing a cotton canopy nitrogen-content estimation model.

**Table 1:** Water and nitrogen treatment settings.

Water treatment	Nitrogen treatment		
	N_1_225 kg ha^-^¹	N_2_300 kg ha^-^¹	N_3_375 kg ha^-^¹
Irrigation level W_1_(375 mm)	W_1_N_1_	W_1_N_2_	W_1_N_3_
Irrigation level W_2_(450 mm)	W_2_N_1_	W_2_N_2_	W_2_N_3_
Irrigation level W_3_(525 mm)	W_3_N_1_	W_3_N_2_	W_3_N_3_

**Table 2:** Fertilization and irrigation ratios (%) for cotton under water and fertilizer integration.

Index	Fertilization and irrigation dates (MM-DD)								
	6-12	6-22	6-30	7-09	7-19	7-29	8-6	8-16	8-26
N fertilizer	10	15	15	15	10	10	10	10	5
PK fertilizer	5	10	10	10	15	15	15	15	5
Irrigation ratio	10	10	12	12	12	12	12	10	10

### 2.3 Data acquisition and processing

#### 2.3.1 Determination of plant nitrogen content

Cotton samples were collected from an experimental field in the Shihezi Reclamation Area of the Eighth Division, Xinjiang Production and Construction Corps, China (44°18′ N, 86°03′ E). The widely cultivated cultivar ‘Jinken 156’ was used. Within each treatment plot, three representative plants were selected from the central mulched rows, ensuring uniform growth and development. Sampling was conducted at four key phenological stages: bud stage (28 June), flowering stage (20 July), boll-setting stage (18 August) and boll-opening stage (4 September). Each plant was separated into stem, leaf and boll fractions. The tissues were enzyme-inactivated at 105°C for 30 min and then oven-dried at 85°C to constant weight; the dry mass of each organ was subsequently recorded. Dried samples were ground, and total nitrogen was determined using the Kjeldahl procedure. Following H2_2_SO_4_–H_2_O_2_ digestion, total N in each organ was quantified using a Kjeldahl analyzer, and the shoot N concentration was calculated (Kimberly and Roberts, 1905).

#### 2.3.2 Acquisition of remote-sensing imagery

A DJI Phantom 4 UAV equipped with a multispectral sensor was deployed to synchronously capture cotton-canopy imagery in five spectral bands centered at 450 nm (B), 560 nm (G), 650 nm (R), 730 nm (RE) and 840 nm (NIR). Flights were conducted under clear, cloud-free conditions between 13:00 and 14:00 h at an altitude of 30 m, with 75 % forward and 75 % side overlap. Subtle fluctuations in lighting and weather during UAV flights can introduce radiometric inconsistencies in multispectral imagery; therefore, radiometric correction is required. In this study, radiometric calibration and image mosaicking were carried out with the professional software Pix4Dmapper. Raw images were captured at one-second intervals throughout each flight, imported into Pix4Dmapper, calibrated to surface reflectance with a radiometric calibration panel, and compiled into an orthomosaic of the study area. The software’s index calculator was then used to derive individual band layers for blue, green, red, near-infrared (NIR), and red-edge wavelengths.

### 2.4 Feature extraction

#### 2.4.1 Vegetation-index derivation

Spectral and textural datasets were extracted from pre-processed UAV imagery. Spectral variables comprised reflectance in five bands—B, G, R, RE and NIR—together with derivative indices computed from their combinations. Because ratio-based vegetation indices normalize band reflectance and thus minimize illumination and background effects, twelve such spectral indices were adopted following earlier studies (Burns et al., 2022; He et al., 2016; Xu et al., 2023), Their formulae and references are listed in **Table 3**.

**Table 3:** Vegetation index calculation formulas.

Vegetation index	Calculation formula	Reference
GRVI	GRVI=(R_green-_R_red_)/(R_green_+R_red_)	(Chen et al., 2019)
GOSAVI	GOSAVI=(R_nir_-R_green_)/(R_nir_+R_green_+0.16)	(Gilabert et al., 2002)
NDVI	NDVI=(R_nir_-R_red_)/(R_nir_+R_red_)	(Pei et al., 2023)
OSAVI	OSAVI=(1 + 0.16)*[(R_nir_-R_red_)/(R_nir_+R_red_+0.16)]	(Li T, et al., 2024)
RVI	RVI=R_nir_/R_red_	(Pokhrel et al., 2023)
MSR	MSR=((Rnir/R_red_)-1)/(((R_nir_/R_red_)+1)^0.5^)	(Fan et al., 2024)
SAVI	SAVI=(1 + 0.5)*[(R_nir_-R_red_)/(R_nir_+R_red_+0.5)]	(Cao et al., 2013)
DVI	DVI=R_nir_-R_red_	(Naito et al., 2017)
NDRE	NDRE=(R_nir-_R_rededge_)/(R_nir_+R_rededge_)	(Gu et al., 2024)
REOSAVI	REOSAVI=(1 + 0.16)*(R_nir_-R_rededge_)/(R_nir_+R_rededge_+0.16)	(Cao et al., 2013)
RETVI	RETVI=0.5*(120*R_nir_-R_red_)-200*(R_rededge_-R_red_)	(Cao et al., 2013)
NDRE_GOSAVI	NDRE_GOSAVI=NDRE/GOSAVI	(Cao et al., 2013)

#### 2.4.2 Texture-feature extraction

Texture metrics characterize the spatial distribution and variability of pixel values, thereby capturing surface properties and canopy spatial structure. Eight Grey-Level Co-occurrence Matrix (GLCM)statistics—mean, variance (Var), dissimilarity (Dis), entropy (Ent), homogeneity (Hom), correlation (Cor), contrast (Con) and second-moment (Sm)—were computed for each of the five bands, producing 40 texture variables. The calculation formulas of each texture feature are given in the reference (Zhou et al., 2021). Texture variables were extracted in Environment for Visualizing Images (ENVI) 5.3 using the grey-level co-occurrence matrix (GLCM) algorithm, a second-order statistical approach.

### 2.5 Feature selection and model development

#### 2.5.1 Elastic Net−based feature selection

Elastic Net is a regularized linear regression technique that combines the characteristics of the least absolute shrinkage and selection operator (LASSO, the L1 penalty) and ridge regression (the L2 penalty). By including both an L1 penalty (which induces sparsity) and an L2 penalty (which induces shrinkage), Elastic Net simultaneously performs feature selection and coefficient estimation within a single modeling step. It retains the ability of Lasso to zero-out certain feature coefficients (effectively removing those features from the model) while also leveraging the stabilizing effect of Ridge on coefficient sizes, especially for correlated features. The Elastic Net objective function adds the L1 and L2 penalty terms to the ordinary least squares loss. For predictor matrix and response vector, the Elastic Net finds coefficient vector β by minimizing a penalized sum of squared errors, as shown in Equation 1:


(1) $$min\beta \frac{1}{2}{\left\|Y-X_{\beta }\right\|}_{2}^{2}+\alpha \rho {\left\|\beta \right\|}_{1}+\frac{\alpha (1-\rho )}{2}{\left\|\beta \right\|}_{2}^{2}$$

In this equation, Y denotes the output vector, X is the input feature matrix, β is the vector of feature weights, and α and ρ are regularization parameters. The parameter ρ controls the relative contribution of L1 and L2 regularization. When ρ = 0, only L2 regularization is applied, while when ρ = 1, only L1 regularization is used. The SHAP regularization terms, with the weighted combination of L1 and L2 penalties determined by ρ.

#### 2.5.2 Boruta–SHAP-based feature selection

Boruta-SHAP is a wrapper-based feature-selection algorithm. The workflow comprises four stages. First, shadow features are generated by randomly permuting each original predictor, thereby removing any real association between those variables and the response. Second, an Extreme Gradient Boosting (XGBoost) model is trained to compute feature-importance scores, and the largest importance value among all shadow features is taken as the reference threshold. Third, any original feature whose importance falls significantly below this threshold is labeled “unimportant” and eliminated from the candidate set. Fourth, all shadow features are discarded and the procedure is iterated until every predictor has been decisively classified (Sebastián and González-Guillén, 2024). In this study, XGBoost serves as the importance evaluator within the Boruta-SHAP framework, allowing us to identify the most informative variables for estimating cotton nitrogen content in arid regions.

#### 2.5.3 Development of the inversion model

To accurately predict the cotton PNC of cotton under subsurface drip irrigation in arid regions, this study employs six machine learning models with strong nonlinear fitting capabilities: three tree based ensemble models (RF, GBDT, XGB) and three kernel based models (SVR, KRR, GPR).RF integrates predictions by constructing numerous independent decision trees, effectively mitigating overfitting and enhancing model stability (Belgiu and Drăguţ, 2016). GBDT iteratively refines decision tree models by learning from residuals at each stage, progressively reducing errors and enhancing predictive performance (Zhang and Jung, 2020). XGB applies a boosting strategy similar to GBDT but incorporates more stringent regularization terms and a weighted optimization process to further enhance prediction accuracy (Yusoff et al., 2025). SVR constructs a maximum margin regression hyperplane in a high-dimensional space, reducing sensitivity to outliers and enhancing the model’s generalization capability (Guo et al., 2021). KRR leverages kernel mapping and ridge regression’s regularization characteristics to effectively mitigate overfitting and achieve superior generalization performance (Campos-Taberner et al., 2016). GPR applies a Gaussian prior in function space and updates the posterior distribution using observational data, effectively quantifying prediction uncertainty while excelling in capturing complex data patterns (Zhu et al., 2023).

The choice of hyperparameter optimization strategy was carefully tailored to each algorithm’s characteristics and computational demands. Bayesian optimization was applied only to RF and XGB because recent agricultural remote sensing studies show that these ensemble learners, with many interdependent hyperparameters and a search landscape without a single convex optimum, benefit most from advanced search strategies. All remaining algorithms were tuned by an exhaustive grid search combined with five-fold cross validation, a decision supported by theoretical analyses and empirical tests. GBDT already uses sequential boosting and shrinkage that provide inherent regularization, which limits the added value of Bayesian optimization. The convex parameter spaces of SVR and KRR can be explored efficiently with systematic grid search. For GPR, training time rises roughly with the cube of the sample size, yet our trials indicated only marginal accuracy gains from a more complex search, so the conventional grid search approach was adopted. All models were implemented in Python 3.7, with training and validation performed using cross-validation.

#### 2.5.4 Model performance evaluation

This study employs the coefficient of determination (R²), root mean square error (RMSE), and mean absolute error (MAE) as the primary metrics to assess model accuracy (Liu et al., 2021). The coefficient of determination (R²) quantifies the model’s explanatory power over data variability, with values closer to 1 indicating a better fit. Root mean square error (RMSE) measures the deviation between predicted and actual values, with smaller values indicating higher prediction accuracy. Mean absolute error (MAE) quantifies the average absolute prediction error, offering an intuitive measure of model performance; smaller values reflect higher prediction accuracy and reduced bias from actual values. The formulas for calculating these evaluation metrics are given below (Equations 2–4), and a comprehensive analysis of these metrics allows for a thorough evaluation of the prediction performance and stability of various models, ensuring the reliability and validity of the findings. All evaluation metrics in this study were implemented within the Python 3.7 environment.


(2) $$R^{2}=1-\frac{\sum_{i=1}^{n}{\left(y_{i}-{\hat{y}}_{i}\right)}^{2}}{\sum_{i=1}^{n}{\left(y_{i}-\bar{y}\right)}^{2}}$$

(3) $$\text{RMSE}=\sqrt{\frac{1}{n}\sum_{i=1}^{n}{\left(y_{i}-{\hat{y}}_{i}\right)}^{2}}$$

(4) $$\text{MAE}=\frac{1}{n}\sum_{i=1}^{n}\left|y_{i}-{\hat{y}}_{i}\right|$$

#### 2.5.5 Rationale for cotton sample selection

The Eighth Division of the Xinjiang Production and Construction Corps is characterized by a typical arid to semi-arid climate, where cotton is extensively cultivated under plastic-mulched drip irrigation. The cotton cultivar ‘Jinken 156’ selected for this study is widely grown and well-adapted to local agro-climatic conditions. By implementing varying irrigation and nitrogen treatments, this research aims to accurately estimate nitrogen nutrition status in cotton grown under drip irrigation conditions in arid regions, thus providing scientific guidance for precise fertilization and irrigation management practices.

All cotton samples used in this research were independently cultivated and managed by the College of Hydraulic and Civil Engineering of Xinjiang Agricultural University and the Xinjiang Academy of Agricultural and Reclamation Sciences. No third-party purchases were involved; therefore, there are no associated receipts or purchase documents.

## 3 Results and analysis

### 3.1 Temporal trend of nitrogen content in plastic-mulched, drip-irrigated cotton

**Figure 2:**
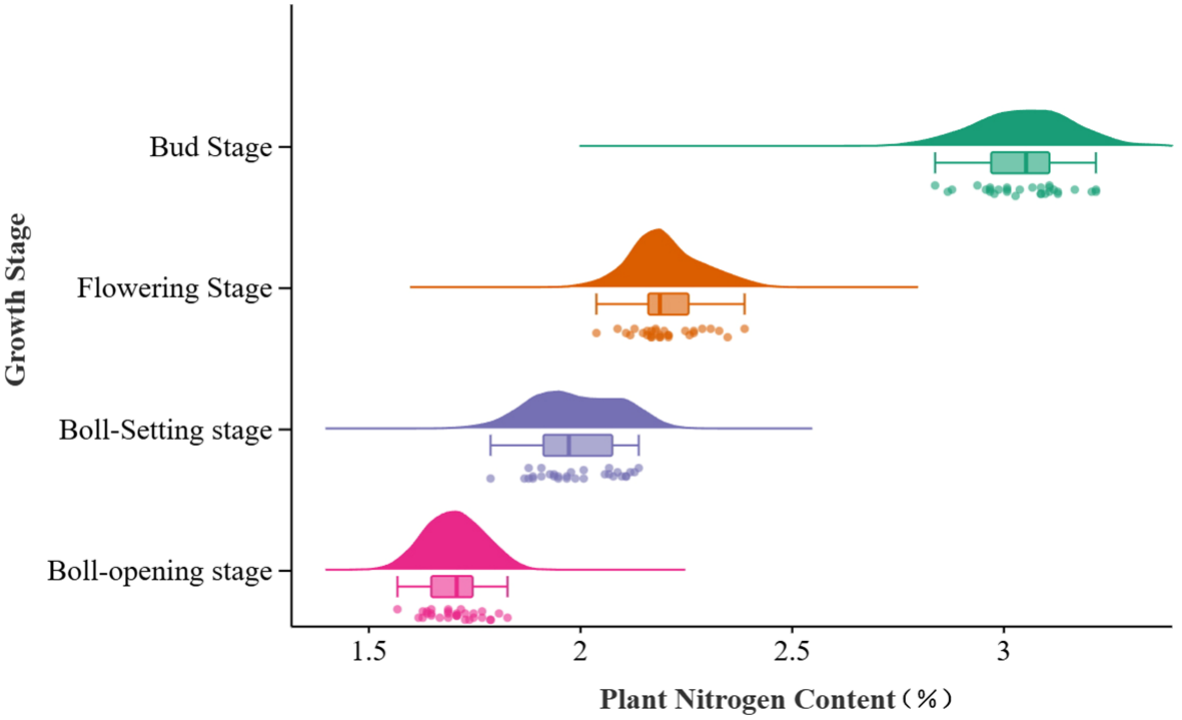
Distribution of cotton plant nitrogen concentration by growth stage. Distribution of plant nitrogen content across four cotton growth stages using violin and box plots.

The trend of nitrogen content (PNC) in drip-irrigated cotton under plastic mulch in arid regions is illustrated in **Figure 2** and **Table 4**. As the growing season progresses, PNC clearly decreases, with the highest nitrogen content observed at the bud stage (2.84%–3.22%) and the lowest at boll-opening (1.57%–1.83%). This suggests that cotton absorbs and accumulates nitrogen more effectively in early growth stages, with this ability weakening in later stages. Regarding data variability, the coefficient of variation (CV) at boll-setting is the highest at 4.76%, reflecting significant variation in nitrogen concentration between individuals at this stage. The CV at boll-opening is lower at 3.58%, suggesting that nitrogen content becomes more consistent among individuals during maturation. Additionally, the small sample standard deviation and variance suggest a low degree of dispersion in PNC, meaning that PNC is uniformly distributed across the sample. Field measurements of PNC delineate the temporal dynamics of nitrogen uptake in cotton cultivated under typical water- and fertilizer-management regimes in arid regions, while providing indispensable ground-truth data for calibrating and validating remote-sensing retrieval models. Given the marked stage-dependent variability and pronounced inter-individual heterogeneity of PNC, remote-sensing features should be exploited to estimate nitrogen status at each phenological stage, thereby laying a robust foundation for subsequent spatial monitoring and analysis.

**Table 4:** Nitrogen content statistics of cotton plants at different growth stages.

Growth stage	Sample size	Minimum	Maximum	Standard deviation	Variance	Coefficient of variation
Bud	30	2.84	3.22	0.11	0.01	3.23
Flowering	30	2.04	2.39	0.07	0.06	3.56
Boll-Setting	30	1.79	2.14	0.09	0.08	4.76
Boll-opening	30	1.57	1.83	0.06	0.03	3.58

### 3.2 Results of Elastic Net-based feature selection

**Figure 3:**
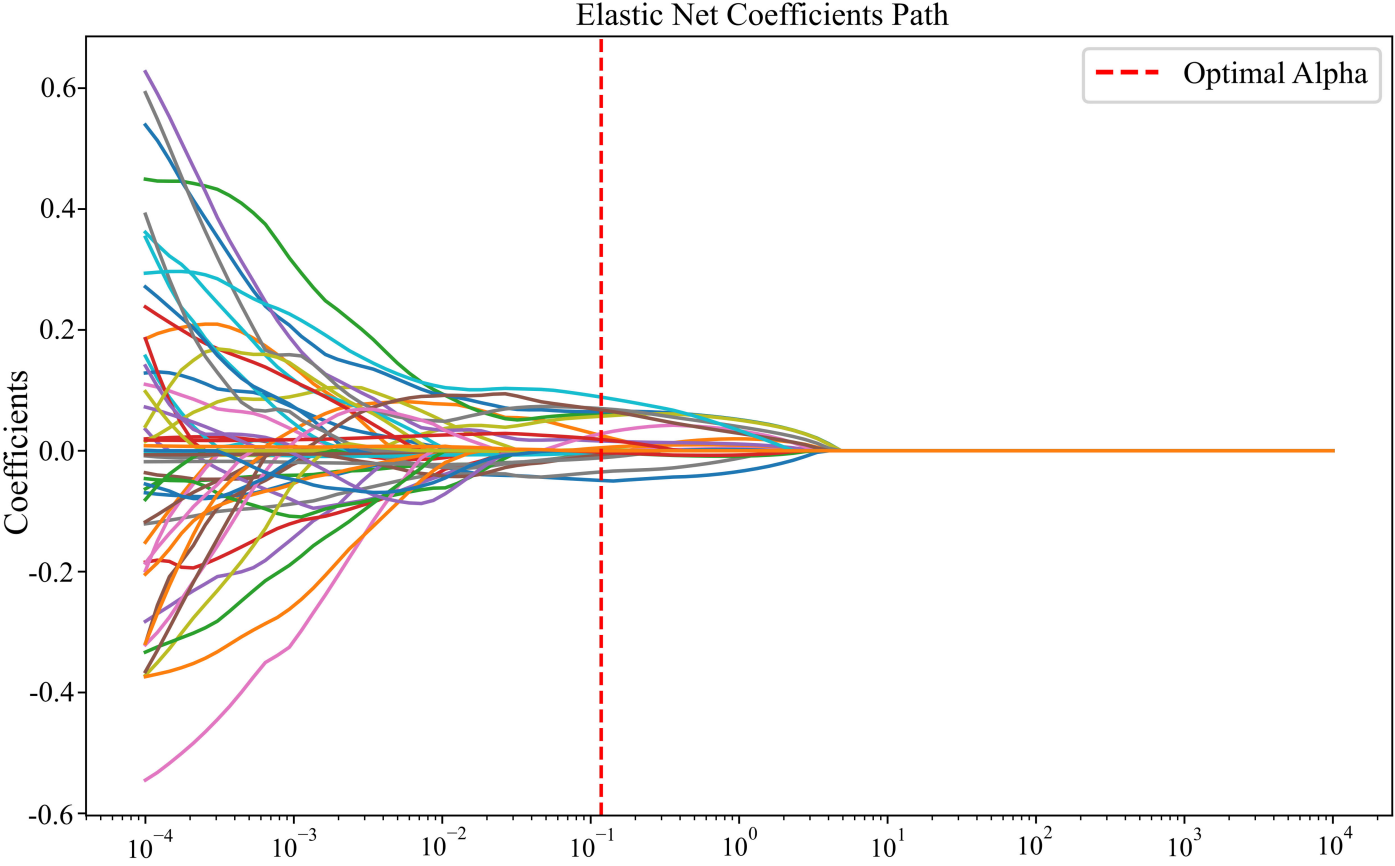
Coefficient path plot. Coefficient trajectories of selected features under different alpha values in the Elastic Net model. The red dashed line indicates the optimal regularization parameter.

**Figure 4:**
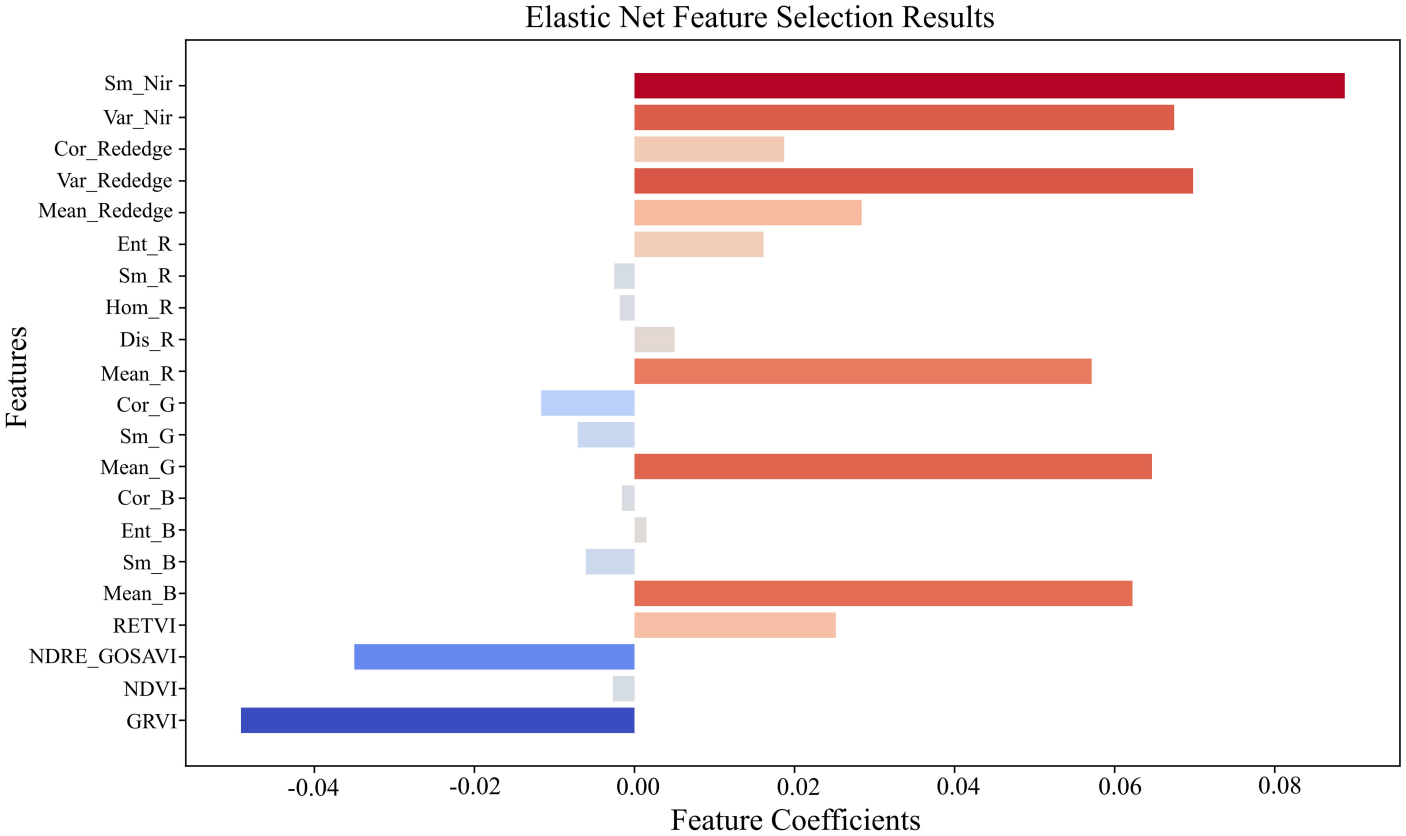
Feature-importance visualization. Feature importance visualization from the Elastic Net model, showing the regression coefficients of selected variables.

To preliminarily identify spectral–textural predictors sensitive to cotton PNC in drip-irrigated cotton under plastic mulch in arid regions, 12 vegetation indices and 40 texture features were incorporated into an Elastic Net model with five-fold cross-validation. The optimal hyper-parameter combination (α = 0.12, l1_ratio = 0.10) indicates that 90% of the regularization weight is assigned to the L2 term, favoring enhanced model stability while retaining partial feature-selection capability. As summarized in **Table 5**, the procedure ultimately retained 21 influential variables that exert a significant effect on PNC. The Elastic Net model attained an RMSE of 0.11 and an R² of 0.95 on the training data, indicating an excellent fit; its performance on the test set was even stronger (RMSE = 0.09, R² = 0.95), confirming the model’s robust generalization capability. The coefficient‐path plot (**Figure 3**) shows that, as the regularization strength (α) increases, most coefficients gradually shrink toward zero, demonstrating that the penalization effectively suppresses redundant variables. The feature‐importance diagram (**Figure 4**) further visualizes each variable’s contribution to PNC, highlighting Sm_Nir and Var_Nir as the most influential predictors, while GRVI and NDRE_GOSAVI display pronounced negative associations. In this figure, the color hue indicates the direction of the regression coefficients—red bars represent positive correlations, and blue bars denote negative correlations—while the color intensity reflects the absolute magnitude of each coefficient, with darker shades indicating greater importance. These quantitative relationships establish a solid foundation for subsequent feature intersection analyses.

**Table 5:** Variables selected by Elastic Net and their regression coefficients.

Feature variable	Regression coefficient	Feature variable	Regression coefficient
NDVI	-0.0027	Cor_B	-0.0016
NDRE_GOSAVI	-0.035	Mean_G	0.0647
RETVI	0.0252	Sm_G	-0.0071
Mean_B	0.0622	Cor_G	-0.0117
Sm_B	-0.0061	Mean_R	0.0571
Sm_Nir	0.0888	Dis_R	0.005
Ent_B	0.0015	Hom_R	-0.0018
Sm_R	-0.0025	Ent_R	0.0161
Mean_Rededge	0.0284	Var_Rededge	0.0698
GRVI	-0.0492	Cor_Rededge	0.0187
Var_Nir	0.0674		

### 3.3 Boruta–SHAP-based feature selection

**Figure 5:**
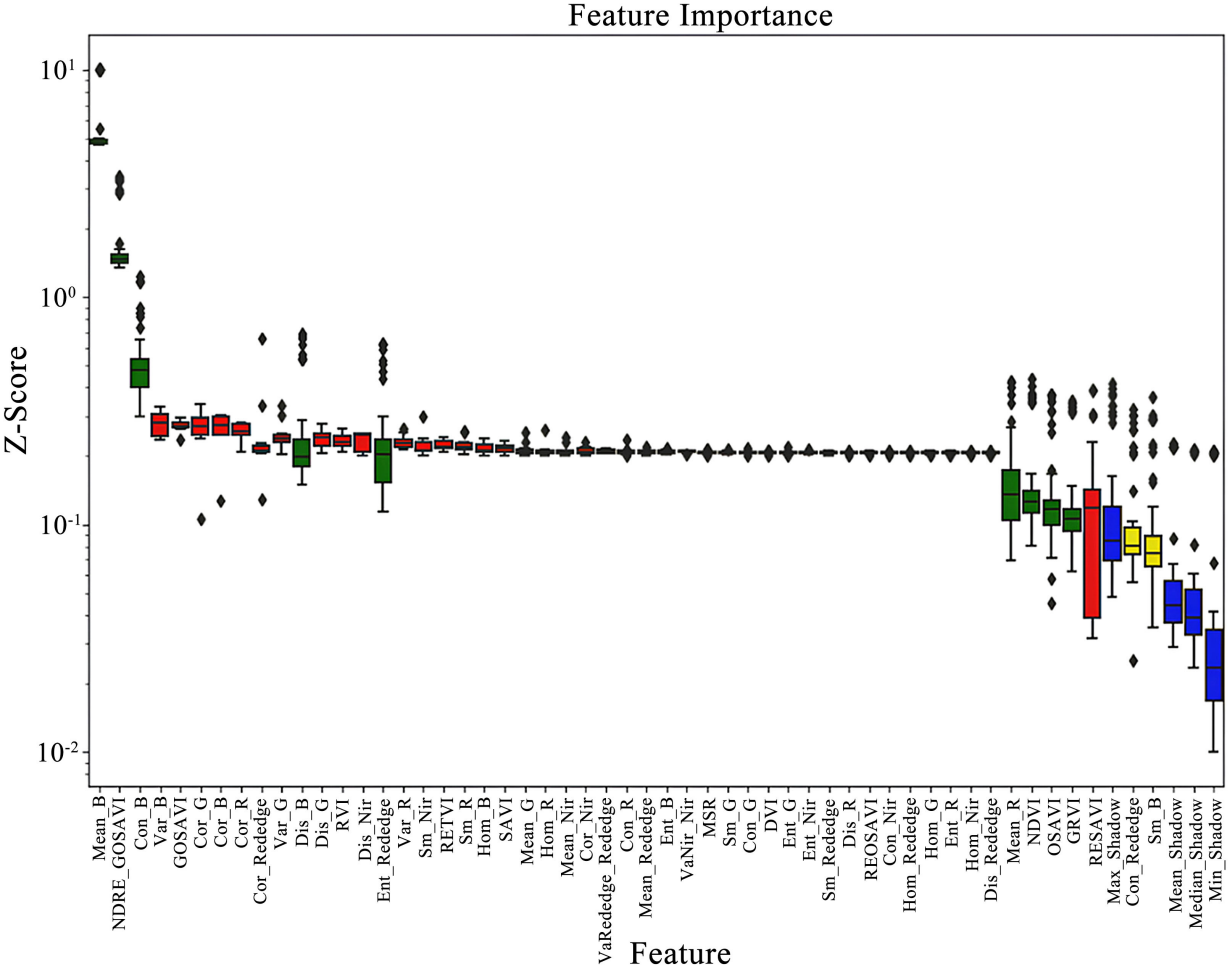
Scoring results presented. Feature importance scores obtained from the Boruta–SHAP method, presented as Z-score distributions across all variables.

Twelve pre-extracted vegetation indices and forty texture features were supplied to the Boruta–SHAP model as input variables. The algorithm calculated Z-scores for the 52 candidate predictors, ranked their importance for estimating cotton PNC in drip-irrigated, plastic-mulched cotton grown in arid regions, and selected the informative variables. In the output, shadowMin, shadowMean, and shadowMax denote the minimum, mean, and maximum Z-scores of the shadow features, respectively. According to XGBoost impurity-based importance (**Figure 5**), the Boruta–SHAP procedure identified nine features—NDRE_GOSAVI, Mean_B, Var_R, Mean_R, NDVI, OSAVI, GRVI, Dis_G, and Con_B—as decisive contributors to PNC prediction. The variables Con_Rededge and Sm_B, whose Z-scores approached shadowMax, were retained as “tentative” features for further scrutiny. To enhance interpretability, **Figure 5** distinguishes importance classes by color: green denotes “important” features, red indicates “unimportant,” yellow marks “tentative,” and blue represents shadow features that provide the reference baseline. This selection strategy reliably isolates informative remote-sensing variables while attenuating noise during subsequent model construction.

### 3.4 Integrated feature selection combining Elastic Net and Boruta–SHAP

**Figure 6:**
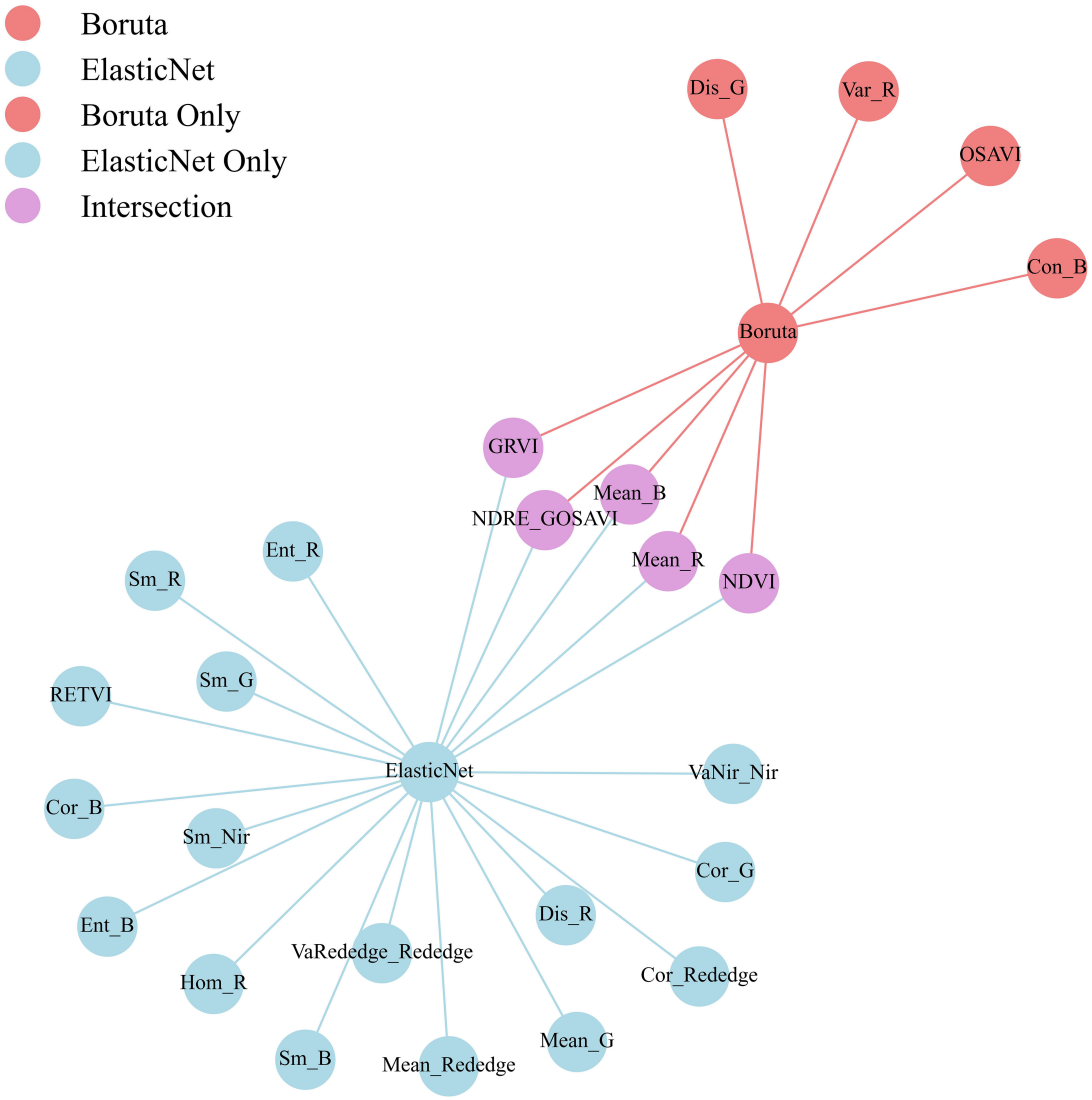
Integrated feature selection based on boruta and elastic net. Integrated feature selection results based on Boruta and Elastic Net. Variables shared by both methods (purple) represent the final set of key predictors for nitrogen estimation.

To further enhance the accuracy and stability of variable selection, this study adopts the intersection of feature variables identified by both Elastic Net and Boruta–SHAP. This approach effectively eliminates redundant and noisy features, ensuring that the selected variables remain significant across different selection mechanisms, thus improving the reliability of feature selection and the robustness of the model. The final 5 selected feature variables, as shown in **Figure 6**, are Mean_B, Mean_R, NDRE_GOSAVI, NDVI, and GRVI, which comprise the final set of sensitive predictors used for PNC prediction in drip-irrigated cotton under plastic mulch in arid regions.

### 3.5 Performance evaluation of the integrated-feature inversion model

**Figure 7:**
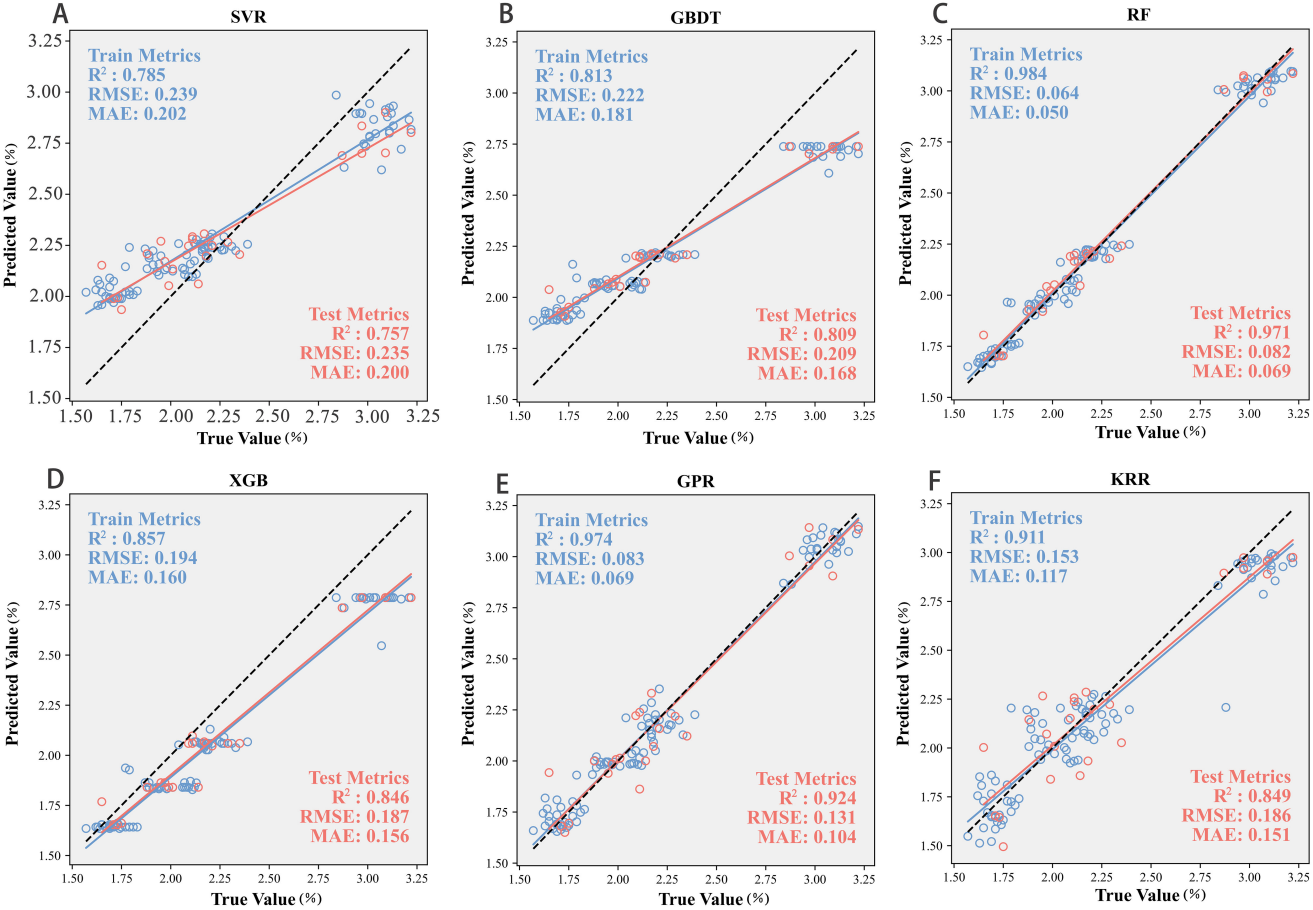
Scatter relationship between measured and predicted values across models. **(A)** Support Vector Regression (SVR), **(B)** Gradient Boosting Decision Tree (GBDT), **(C)** Bayesian Optimized Random Forest (RF), **(D)** Extreme Gradient Boosting (XGB), **(E)** Gaussian Process Regression (GPR), and **(F)** Kernel Ridge Regression (KRR).

**Figure 8:**
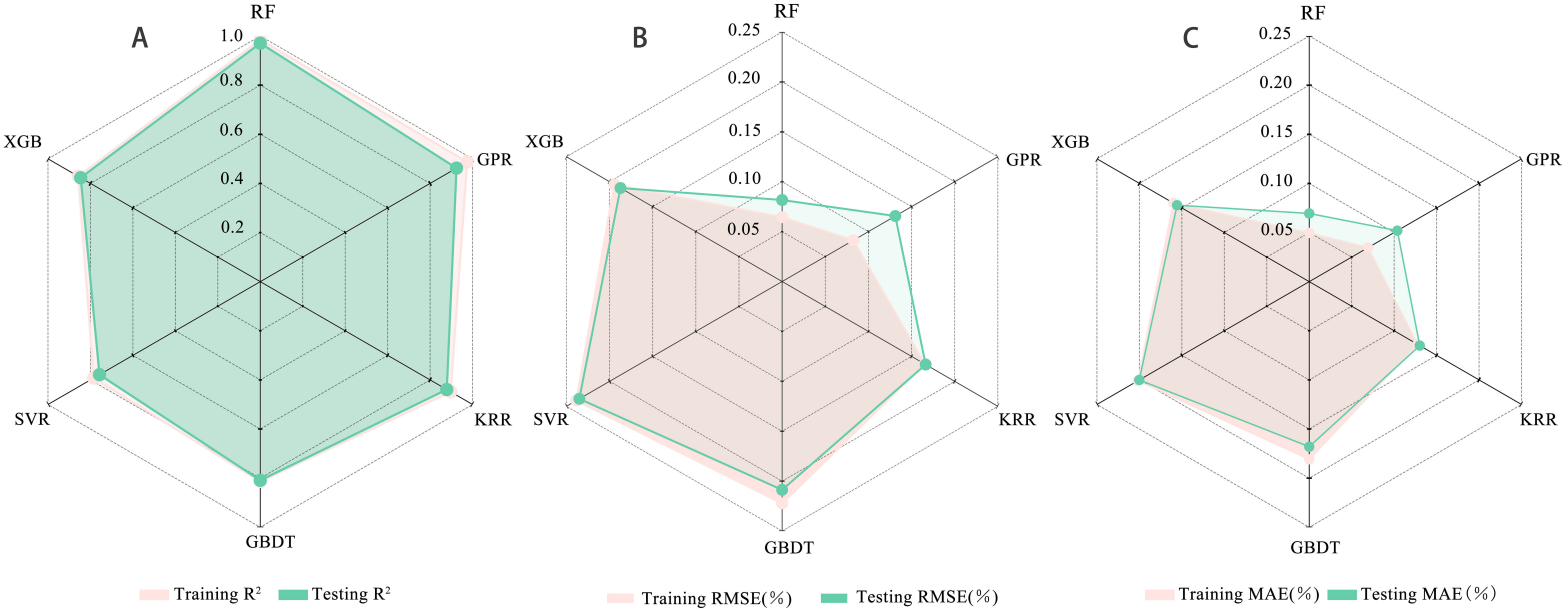
Visual representation of model evaluation indices. **(A)** Coefficient of determination (R²), **(B)** Root mean square error (RMSE), and **(C)** Mean absolute error (MAE).

Based on the results of the multi-tier feature selection, the identified sensitive features were used as input variables, and cotton PNC was set as the response variable. Six machine learning models—RF, GBDT, XGB, SVR, KRR, and GPR—were developed and evaluated using R², RMSE, and MAE to assess the fitting accuracy and generalization performance on both training and test datasets (**Table 6**). To systematically analyze differences in model applicability across algorithm categories, the models were grouped into decision tree–based and kernel–based types for comparative evaluation. The results are presented in **Figures 7**, **8**.

**Table 6:** Predictive performance of various machine-learning models for PNC.

Models	Training set			Test set		
	R^2^	RMSE%	MAE%	R^2^	RMSE%	MAE%
RF	0.98	0.06	0.04	0.97	0.08	0.06
GBDT	0.81	0.22	0.18	0.80	0.20	0.16
XGB	0.85	0.19	0.16	0.84	0.18	0.15
SVR	0.78	0.23	0.20	0.75	0.23	0.20
KRR	0.91	0.15	0.11	0.84	0.18	0.15
GPR	0.97	0.08	0.06	0.92	0.13	0.10

Among the tree-based ensemble models, RF exhibited the best overall performance. It achieved R² values of 0.98 and 0.97 for the training and test sets, respectively, with RMSE values of 0.06 and 0.08 and MAE values of 0.04 and 0.06, demonstrating excellent fitting accuracy and generalization capability. This superiority can be attributed to RF’s ensemble learning strategy and random feature-sampling mechanism, which effectively reduce overfitting. In contrast, XGB and GBDT showed lower prediction accuracy. XGB yielded R² values of 0.85 and 0.84, with RMSE values of 0.19 and 0.18, indicating moderate stability but limited accuracy. GBDT performed even worse, with R² values of 0.81 and 0.80 and RMSE values of 0.22 and 0.20, suggesting limited adaptability to the current dataset and a need for further parameter tuning. Within the kernel-based models (SVR, KRR, GPR), GPR demonstrated the strongest performance. It achieved R² values of 0.97 (training) and 0.92 (test) with correspondingly low RMSE and MAE, ranking second only to RF and confirming its capacity to capture nonlinear relationships while providing uncertainty estimates. KRR performed well on the training set (R² = 0.91) but dropped to 0.84 on the test set, with RMSE and MAE increasing to 0.18 and 0.15, indicating a tendency toward overfitting. SVR recorded the lowest accuracy among all six models, with R² values of 0.78 and 0.75 and the highest RMSE and MAE (0.23 and 0.20), reflecting limited suitability for the present data conditions, possibly due to its sensitivity to high-dimensional and noisy inputs.

Based on the performance differences among models in the PNC prediction task, tree ensemble models generally outperform kernel methods. In particular, the RF model achieves high fitting accuracy and shows strong generalization ability, underscoring its stability and applicability in complex nonlinear modeling scenarios. Although GPR performs slightly worse, its capacity for uncertainty estimation provides substantial added value in application oriented contexts. By contrast, SVR displays poor stability and accuracy in prediction, indicating that it is unsuitable for this dataset.

**Figure 9:**
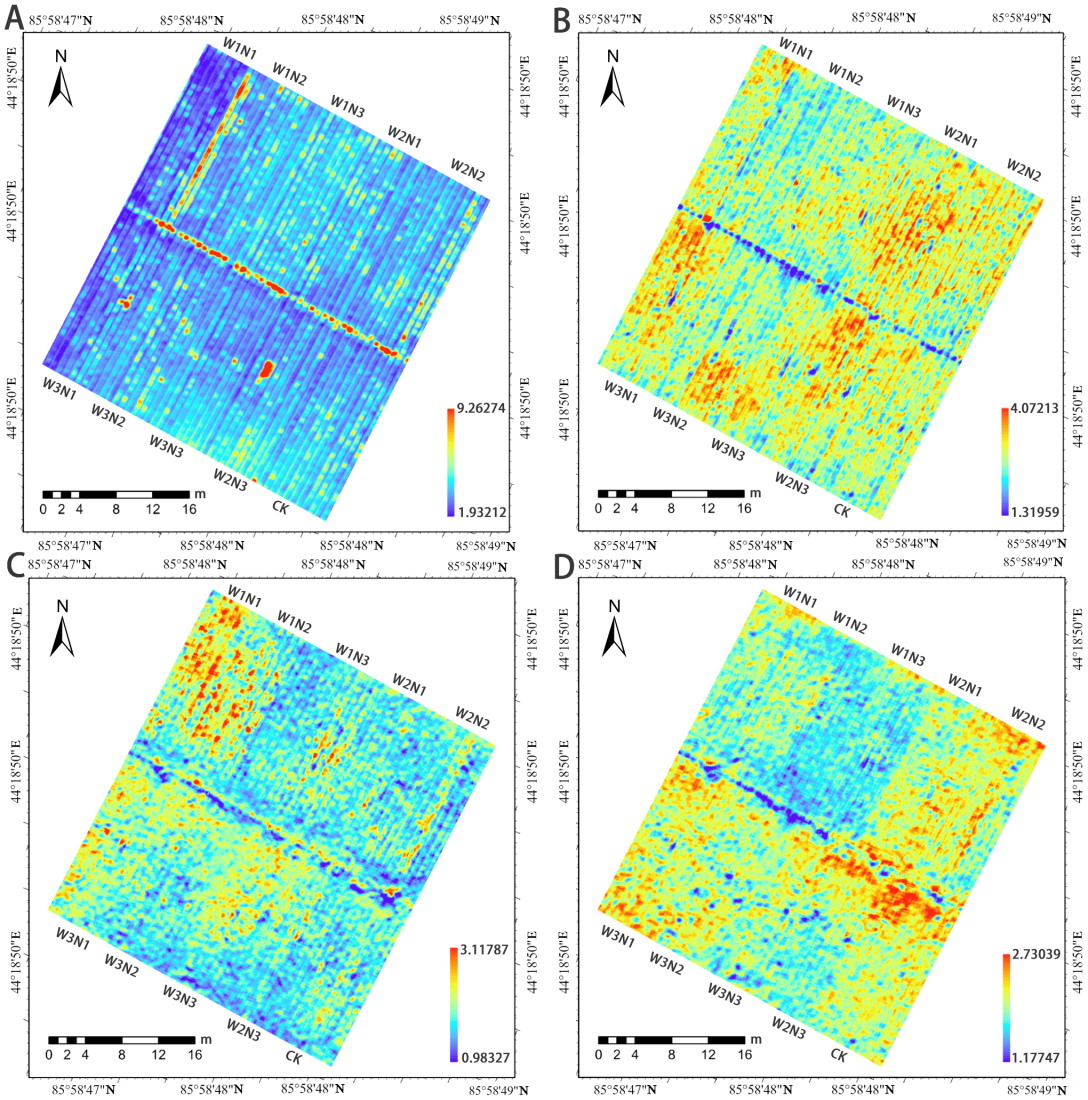
Spatiotemporal inversion map of PNC during different growth stages of cotton. **(A)** Bud stage, **(B)** Flowering stage, **(C)** Boll-setting stage, and **(D)** Boll-opening stage.

A comprehensive comparison analysis identified the RF model as the optimal PNC estimation model for cotton. **Figure 9** shows the spatiotemporal distribution of estimated PNC across four growth stages based on this model. The analysis reveals a general decline in cotton PNC across growth stages, with values ranging from 1.93% to 3.42% at the bud stage, 1.31% to 2.39% at flowering, 1.17% to 2.21% at boll-setting, and 0.98% to 1.78% at boll-opening. Under identical nitrogen application rates, the highest cotton PNC is observed at the W2 irrigation level (1.67%–3.22%), while PNC at W1 and W3 decrease to 1.57%–3.11% and 1.69%–2.94%, respectively. This suggests that increased irrigation enhances cotton’s nitrogen absorption. However, as plant biomass increases, nitrogen dilution becomes evident, leading to a decrease in nitrogen concentration in cotton plants Under the same irrigation conditions, PNC differs across nitrogen application gradients. N1 (1.24%–2.97%) and N3 (1.31%–3.11%) show lower values than N2 (1.33%–3.28%), indicating that increasing nitrogen application significantly boosts cotton nitrogen content. Furthermore, an optimal nitrogen level promotes nitrogen uptake and accumulation in cotton, whereas excessive nitrogen may inhibit absorption and utilization. This result aligns with the previous analysis of measured data, confirming that the model provides accurate predictions of cotton PNC.

## 4 Discussion

### 4.1 Effect of feature selection on PNC estimation accuracy

In this study, five key features (NDVI, GRVI, NDRE_GOSAVI, Mean_R, and Mean_B) were identified through a multi-stage selection pipeline that combined Elastic Net and Boruta SHAP, and these features were found to be strongly associated with crop nitrogen status. Previous studies have shown that vegetation indices such as NDVI, GRVI, and NDRE_GOSAVI effectively reflect leaf nitrogen content and plant growth status. At the vegetation index level, consistent correlations between multiple vegetation indices and cotton leaf nitrogen content were reported by Yin et al (Yin et al., 2022). In addition, GRVI and GNDVI were shown by Maresma et al. in a maize experiment to discriminate significantly among nitrogen treatments, illustrating the high diagnostic power of green red normalized indices for nitrogen monitoring (Maresma et al., 2016). At the texture level, contributions of the standard deviation of texture features derived from gray level co-occurrence matrices and of color features extracted from UAV RGB images to nitrogen content prediction in cotton were demonstrated by Kou et al (Kou et al., 2022). These results further support the scientific rigor and effectiveness of the integrated feature selection strategy employed in the present study. At the feature-selection stage, feature sparsity and variable screening are achieved by simultaneously applying L1 and L2 regularization within Elastic Net, effectively reducing multicollinearity among remote-sensing features. Compared with earlier studies that relied solely on LASSO or ridge regression, this approach retains important correlated variables more effectively (Chen et al., 2025). Twenty-one key variables were selected by the Elastic Net method. Their predictive power showed strong generalization, with R² values of 0.95 for the training set and 0.95 for the test set, confirming the effectiveness of this approach for remote sensing feature selection. In contrast, Boruta-SHAP creates shadow features and combines them with an XGBoost model to evaluate feature importance, thereby accurately identifying variables that significantly influence PNC within the high dimensional feature space (Abdelwahed et al., 2022). Within the present study, nine significant and two tentative features were selected by Boruta SHAP. Their intersection with the Elastic Net output yielded a core set of five variables—Mean_B, Mean_R, NDRE_GOSAVI, NDVI, and GRVI. These variables encompass both spectral indices and texture metrics, enabling a more comprehensive representation of canopy nitrogen variation. Accordingly, the integrated selection strategy that combines Elastic Net with Boruta SHAP facilitates the extraction of robust, representative nitrogen-sensitive features and enhances the accuracy and reliability of the cotton nitrogen estimation model.

### 4.2 Effect of machine-learning models on PNC estimation

Based on the comparative results of six machine learning algorithms, the Bayesian optimized Random Forest (RF) performed best in estimating cotton PNC, achieving high accuracies of R² = 0.98/0.97 and RMSE = 0.06/0.08 on the training and test sets, respectively. Notably, the superiority of RF is evident not only over the unoptimized baseline models but also over Extreme Gradient Boosting (XGB), which was tuned with the same Bayesian optimization strategy. Although both belong to the ensemble tree family, RF still exceeds XGB on the test set by 0.13 in R² (ΔR² = 0.13). This gap indicates that RF’s advantage arises primarily from its structural properties, namely its use of random feature subspaces and ensemble averaging to handle high dimensional and collinear features, rather than from hyperparameter tuning alone.

Similar research showed that Lu et al. found in maize nitrogen estimation using agricultural remote sensing that RF (R² = 0.93) outperformed XGB (R² = 0.87) (Lu et al., 2025). Khodjaev et al. further noted that, in multi-environment wheat-yield prediction tasks, Random Forest exhibited greater robustness to feature redundancy (Khodjaev et al., 2025). In conjunction with the hierarchical feature-selection strategy adopted in the present study, these findings suggest that RF and Bayesian optimization work synergistically, conferring unique advantages in handling the high-dimensional data generated by UAV remote sensing. Several recent investigations spanning two successive seasons or multiple geographic sites have consistently verified that RF models achieve high precision (R² ≈ 0.85 – 0.95) for cotton nitrogen estimation, indicating robust generalizability over time and space (Li M, et al., 2024). Gaussian Process Regression (GPR) ranked second, with R² = 0.97/0.92, and its strengths lie in its probabilistic framework and built-in uncertainty quantification. However, its high computational cost, coupled with only marginal accuracy gains over the grid-search-tuned variant employed in this study, restricts its practical applicability. By contrast, RF preserves high accuracy while offering excellent computational efficiency, a combination that is critical for scalable precision-agriculture applications. Support Vector Regression (SVR) performed the worst; this observation concurs with Zhang et al. in a wheat-nitrogen study, underscoring SVR’s limitations in coping with feature collinearity and spectral noise (Zhang et al., 2024). The performance disparities among these models further indicate that model selection should jointly consider optimization strategies and the algorithm’s intrinsic compatibility with data characteristics; for UAV-based remote-sensing datasets, tree-based ensemble models such as RF demonstrate clear advantages over kernel methods in dealing with multicollinearity and complex nonlinear structures.

### 4.3 Effect of plastic-mulched drip context on PNC accuracy

In plastic-mulched, drip-irrigated cotton systems in arid regions, the mulch markedly enhances reflectance in the 450–700 nm visible band, thereby weakening the ability of conventional vegetation indices to characterize canopy nitrogen status (Cheng et al., 2024). To mitigate this interference, the present study developed a multi-level feature-selection framework that jointly identifies spectral and textural features resistant to mulch effects, thereby markedly enhancing model robustness under complex spectral backgrounds. The results indicate that mulch reflectance is strongest at the early growth stage, during which the accuracy of traditional indices declines most sharply. The NDRE avoids the mulch reflectance peak by shifting its sensitive band to 705 to 750 nm. GOSAVI, in contrast, employs a soil adjustment factor to dynamically correct bright or moist backgrounds, and—when coupled with NDRE—further smooths the reflectance steps introduced by the mulch (Cao et al., 2016). Therefore, meticulous radiometric calibration together with the selection of interference resistant indices is essential for reliable nitrogen monitoring in arid regions. Beyond spectral information, textural features also play an indispensable role. Local gray level difference operations can offset overall brightness elevation within limited neighborhoods, thereby attenuating the brightening effect of the mulch. The mulch appears as regular stripes or patches, whereas the cotton canopy exhibits more random and isotropic textures; metrics such as contrast and mean can therefore effectively separate the two structures and reduce spectrally mixed pixels. Furthermore, texture features can sensitively capture subtle differences in canopy structure and nitrogen spatial distribution under arid conditions, thereby further improving the accuracy of nitrogen concentration estimation (Zhang et al., 2024).

Compared with humid or semi-humid ecosystems, the intense radiation, high evapotranspiration, and plastic-mulched drip irrigation characteristic of arid regions substantially increase the complexity of nitrogen monitoring. These environmental factors significantly modify canopy reflectance spectra and cotton nitrogen-uptake dynamics, leading to more severe collinearity and redundancy when features are extracted from multisource remote-sensing data. To address these challenges, the present study combines the strengths of the Elastic Net and Boruta SHAP algorithms to simultaneously mitigate high-dimensional collinearity, feature redundancy, and model interpretability issues. Water scarcity is the principal constraint on agriculture in arid zones, dictating that cotton is commonly managed via coordinated water-and-fertilizer regimes. Accordingly, we designed distinct water–nitrogen coupling treatments and, through multilevel feature selection and machine-learning modeling, evaluated how changes in water and nitrogen supply affect cotton nitrogen status under arid conditions. This method accommodates the compound spectral-texture interference characteristic of plastic-mulched drip systems in arid lands and addresses the common neglect of arid-specific environmental factors in previous studies. Compared with approaches developed for other ecological zones, our study highlights the technical challenges posed by the combined effects of plastic mulching and arid environments and provides a corresponding systematic solution. By explicitly identifying and purposefully resolving these unique technical bottlenecks, this study substantially improves the practicality and predictive accuracy of UAV-based nitrogen-monitoring models in plastic-mulched drip-irrigated cotton fields of arid regions.

### 4.4 Study limitations

1. The experimental data are derived exclusively from plastic-mulched, drip-irrigated cotton fields in arid Xinjiang, where uniform geo-climatic conditions may constrain the model’s ability to generalize to ecologically heterogeneous regions such as the cotton-growing areas of the Yellow River Basin. Future research should collect samples across multiple ecological zones and apply transfer-learning frameworks to improve cross-regional adaptability. Moreover, the small sample size is particularly problematic for kernel-based models, hindering the full exploitation of their inherent advantages.
2. The current study focuses on estimating nitrogen content across the entire growth period but, owing to insufficient temporal resolution with 22–29 day sampling intervals, fails to capture short-term nitrogen dynamics following fertigation events. In addition, the current method has been validated only for a single crop (cotton). Future studies should incorporate more frequent sampling protocols and include a wider array of crop species to provide a more comprehensive evaluation of the method’s general applicability.

## 5 Conclusions

This study, on the basis of UAV remote-sensing imagery, combines a feature-selection approach to filter spectral information and, using the evaluated random forest, builds an estimation model for cotton PNC of drip-irrigated, plastic-mulched cotton, and mainly obtains the following conclusions:

1. Five key feature variables (Mean_B, Mean_R, NDRE_GOSAVI, NDVI, GRVI) were optimally selected from the variables, which can significantly improve cotton PNC prediction accuracy.
2. By comparing the predictive performance of six machine-learning models, it was found that RF achieved R² of 0.98 and 0.97 on the training and test sets, respectively, with RMSE of 0.05 and 0.08, outperforming other models in prediction accuracy and being relatively stable for estimating PNC of plastic-mulched drip-irrigated cotton.
3. Field measurements and inversion results at four key growth stages showed a general declining trend in PNC throughout the cotton growth cycle, indicating pronounced stage-dependent nitrogen uptake dynamics. Water–nitrogen interaction analysis further demonstrated that the W_2_N_2_ treatment (450 mm seasonal irrigation combined with 300 kg N ha^-^¹) sustained the highest PNC (1.83% to 3.22%) across all growth stages, whereas either insufficient or excessive water or nitrogen reduced uptake efficiency. For arid, plastic-mulched, drip-irrigated cotton, we recommend a seasonal irrigation quota of 425 mm to 475 mm and a nitrogen input of 250 kg N ha^-^¹ to 320 kg N ha^-^¹, with cultivar-, soil- and nutrient-specific adjustments to balance yield targets with nitrogen-use efficiency.

## Data Availability

The raw data supporting the conclusions of this article will be made available by the authors, without undue reservation.
